# When at night I go to sleep / Fourteen angels watch do keep^1^

**DOI:** 10.3201/eid1909.AC1909

**Published:** 2013-09

**Authors:** Polyxeni Potter

**Affiliations:** Centers for Disease Control and Prevention, Atlanta, Georgia, USA

**Keywords:** art science connection, emerging infectious diseases, art and medicine, When at night I go to sleep, Fourteen angels watch do keep, The Miracle of Saint Vitus, Saint Willibrord, Franconian painter, bizarre behavior, nodding syndrome, neurologic disorders, about the cover

**Figure:**
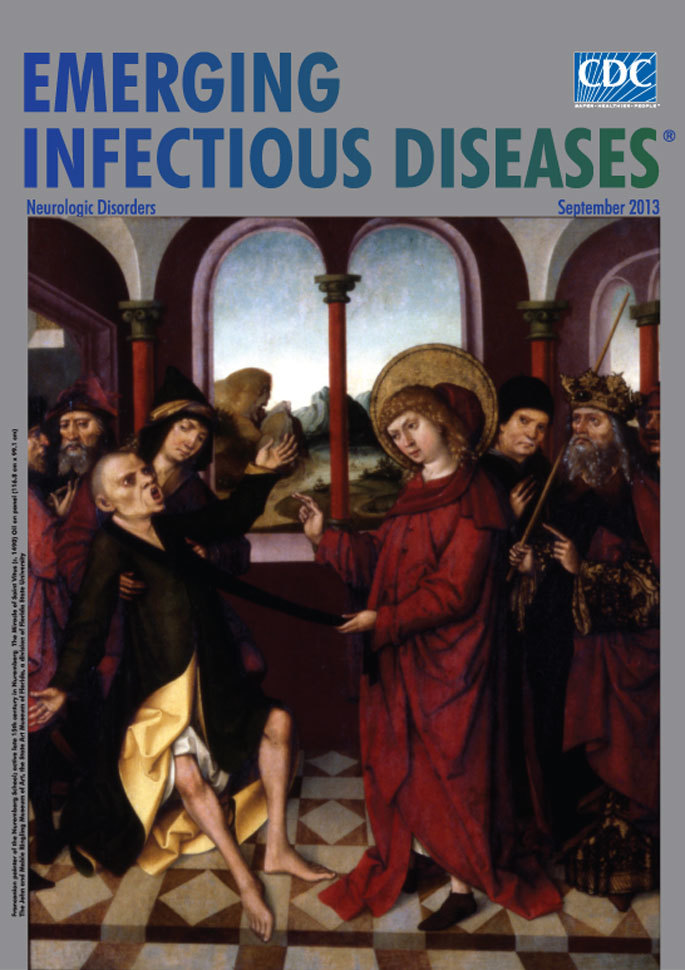
**Franconian painter of the Nuremberg School; active late 15th century in Nuremberg *The Miracle of Saint Vitus* (c. 1490) Oil on panel (116.8 cm × 99.1 cm)** The John and Mable Ringling Museum of Art, the State Art Museum of Florida, a division of Florida State University

Every year, on Whit Tuesday, pilgrims crowd the small town of Echternach, Luxembourg, to join a Dancing Procession, a tradition, legend has it, rooted in miraculous healings as far back as the 8th century at the site of Saint Willibrord’s sarcophagus. The procession, first mentioned in the city archives in 1497, moves rhythmically, three steps forward, two steps back, and slowly―five steps needed to advance one―the folk dancers, four or five abreast, holding on to each other.

Willibrord, known as Saint Witt (Vitus) in Germany and as Saint Guy in France, was an English missionary to Denmark and the Netherlands and the first Bishop of Utrecht, who founded the Benedictine Abbey of Echternach. Word of healings at his graveside spread far and wide: a woman brought there unable to walk left “using her own legs.” A man with fainting spells and tremor in the limbs was cured.

Willibrord became the patron saint of patients with neurologic diseases: paresis, epilepsy, and what was then known as choreomania or the “dancing disease,” an epidemic of strange behavior of crowds. They would suddenly form circles and dance to exhaustion, “their limbs jerked and they collapsed snorting, unconscious and frothing.” This epidemic of uncontrolled dancing spread throughout Germany and the Netherlands. Outbreaks also occurred in France and Britain. “Peasants left their ploughs, merchants their workshops, and housewives their domestic duties to join the wild revels.”

Paracelsus (1493‒1541) described the dancing mania as chorea (Gr. *χορεíα* = dance) Sancti Viti and raised it from superstition to a disorder manifested in three types, one of them, chorea naturalis (arising from physical causes). The dancers were not uniformly hysterical. Some, who exhibited neurologic symptoms of unknown origin, were removed from the procession by the authorities. Others were the relatives and friends of the sick, who danced for the healing of their beloved.

Felix Platter, in his Praxeos Medicae (1602), referred to Saint Vitus’ dance as “that frightening and remarkable though rare disease,” an epidemic in which not just women but men too were afflicted with dancing that went on for weeks. He noted a possible parallel with a peculiar “jumping condition of the limbs,” known to the Arabs and concluded that if this disease is not from the devil, it must come from God himself as punishment.

The term Saint Vitus’ dance became popular after early epidemiologist Thomas Sydenham (1624‒1689) used it in his classic description of acute chorea, “St Vitus dance is a sort of convulsion which attacks boys and girls from the tenth year until they have done growing. At first it shows itself by a halting, or rather an unsteady movement of one of the legs, which the patient drags. Then it is seen in the hand of the same side. The patient cannot keep it a moment in its place, whether he lay it upon his breast or any other part of his body. Do what he may, it will be jerked elsewhere convulsively.”

*The Miracle of Saint Vitus*, on this month’s cover, is an account of chorea-like illness. As the clinical features were heterogeneous, Saint Vitus’ dance became an umbrella term for an assortment of kinetic disorders. In this scene, the unknown painter captures for posterity the frightful plight of a person with neurologic illness. Helpless and misunderstood, he awaits a miracle that will loosen his muscles, unlock his mouth, and steady his limbs. At the same time, the painting sheds light on how a saint and his name might have become intricately connected with the activities in Echternach and elsewhere.

The martyr Saint Vitus lived in Sicily and was mentioned in historical documents as early as the 5th century. He was one of 14 Holy Helpers, grouped together in the Middle Ages, when the Black Death was spreading throughout Europe decimating the population. These saints were pressed to action because of their healing powers against a variety of disorders, from headache and abdominal pain to bubonic plague. Saint Vitus’ feast day was celebrated by dancing before his statue because he was the patron saint of dancers and entertainers. But he was also the protector of those sick with “unsteady step, trembling limbs, limping knees, bent fingers and hands, paralysed hands, lameness, crookedness, and withering body.” The signs and symptoms mimicked the movements of dance.

This Saint Vitus’ early life is clouded in legend. Hagiographic texts describe him as a spiritual and holy child, possessing extraordinary powers, much to the chagrin of his family. During his brief life, he was hounded for his rejection of idolatry and traveled around to escape persecution, all the while performing miracles. He is known for healing Roman Emperor Diocletian’s son, who was thought to be possessed by demons because he twitched uncontrollably. In a singular gesture of ungratefulness the emperor pronounced his son’s cure unholy magic and had Vitus thrown into a cauldron of boiling oil.

*The Miracle of Saint Vitus* was a product of the Nuremberg School, a tradition named after a major artistic and commercial center of the times. The Franconian city, which in its early days had no well-known painter at the helm, at its peak boasted none other than the great Albrecht Dürer. Long and enduring, this artistic tradition encompassed the late Gothic and early Renaissance art of Northern Europe, which was rich and diverse, embracing eastern and western elements and culminating in a Gothic style softened with features from Italian art and the advent of oil painting.

“Realism of particulars,” along with symbolism, characteristic elements of Franconian painting, is at work in this theatrical scene. The architectural details place the viewer in a palatial context with stately columns and marble floor, framed on the right by the emperor himself sporting, along with crown and scepter, regal attire richly trimmed at the sleeve and hemline. The onlookers’ expressions reflect the usual: ignorance, curiosity, suspicion, revulsion, fear, mockery.

The attendants trying to control and stabilize the emperor’s son recall the “fourteen angels” of the lost children’s prayer from Engelbert Humperdinck’s fairy opera Hansel and Gretel. The angels symbolize the Fourteen Holy Helpers providing assistance to the patient, who falls back as his legs give out under him: “Two my head are guarding, / Two my feet are guiding.” Unable to control his body, he is at their mercy: “Two upon my right hand, / Two upon my left hand.” His face distorted, dystonic mouth frozen, eyes transfixed, he is finished. “Two to whom ‘tis given / To guide my steps to heaven.”

At the center of the painting, the young saint outshines the emperor, who seems diminished, secondary, frightened, and helpless, even as he extends the index finger of authority. In this boldly colored room, against the atmospheric perspective beyond the window, the saint is king. He is attentive and focused, one hand touching the patient’s cloth, the other raised in a symbolic gesture.

The modern version of Saint Vitus’ dance, Sydenham chorea, the prototype for an autoimmune-mediated chorea following infectious illness, is still characterized by involuntary movements, which “flit and flow unpredictably from one body part to another.” They can be caused by several infections, among them HIV and tuberculosis.

The epidemiologic features of neurologic disorders are confusing and rival in complexity the cryptic descriptions of the lives of saints. Diseases of the same cause are expressed differently and those with different cause sometimes look the same, a difficulty also encountered often in the study of emerging infections. While in times past those affected sought the answer in theological miracles, now we sometimes look for scientific miracles, or at least statistical ones.

Bizarre behavior, whether frantic or somnolent, is caused by a growing procession of ailments, among them rabies, nodding syndrome, unexplained epidemic epilepsy associated with onchocerciasis, diphtheric polyneuropathy, acute flaccid paralysis, brucellosis, encephalitis, or various fevers of unknown origin. Bizarre behavior still relies for relief on miracles, which today’s science measures as probability (p values). Epidemiologists at a slow but steady pace, generate surveillance data around the globe, three steps forward, two steps back. The hope is to enable patients with neurologic disorders to gain control over their bodies and only dance when they choose to.
